# Characterization of peritoneal cells from cats with experimentally-induced feline infectious peritonitis (FIP) using RNA-seq

**DOI:** 10.1186/s13567-018-0578-y

**Published:** 2018-08-07

**Authors:** Rie Watanabe, Christina Eckstrand, Hongwei Liu, Niels C. Pedersen

**Affiliations:** 1 Center for Companion Animal Health, School of Veterinary Medicine, University of California, Davis, CA USA; 2 Department of Pathology, Microbiology and Immunology, School of Veterinary Medicine, University of California, Davis, CA USA

## Abstract

Laboratory cats were infected with a serotype I cat-passaged field strain of FIP virus (FIPV) and peritoneal cells harvested 2–3 weeks later at onset of lymphopenia, fever and serositis. Comparison peritoneal cells were collected from four healthy laboratory cats by peritoneal lavage and macrophages predominated in both populations. Differential mRNA expression analysis identified 5621 genes as deregulated in peritoneal cells from FIPV infected versus normal cats; 956 genes showed > 2.0 Log_2_ Fold Change (Log_2_FC) and 1589 genes showed < −2.0 Log_2_FC. Eighteen significantly upregulated pathways were identified by InnateDB enrichment analysis. These pathways involved apoptosis, cytokine–cytokine receptor interaction, pathogen recognition, Jak-STAT signaling, NK cell mediated cytotoxicity, several chronic infectious diseases, graft versus host disease, allograft rejection and certain autoimmune disorders. Infected peritoneal macrophages were activated M1 type based on pattern of RNA expression. Apoptosis was found to involve large virus-laden peritoneal macrophages more than less mature macrophages, suggesting that macrophage death played a role in virus dissemination. Gene transcripts for MHC I but not II receptors were upregulated, while mRNA for receptors commonly associated with virus attachment and identified in other coronaviruses were either not detected (APN, L-SIGN), not deregulated (DDP-4) or down-regulated (DC-SIGN). However, the mRNA for FcγRIIIA (CD16A/ADCC receptor) was significantly upregulated, supporting entry of virus as an immune complex. Analysis of KEGG associated gene transcripts indicated that Th1 polarization overshadowed Th2 polarization, but the addition of relevant B cell associated genes previously linked to FIP macrophages tended to alter this perception.

## Introduction

Macrophages are the main host cell supporting FIPV replication in vivo [1]. It is therefore important to study how FIPV infected macrophages respond to infection, because they also mediate the resultant immune/inflammatory responses. FIPV replication appears to be very cell associated throughout the disease course and there appears to be no discernable cell-free viremia [1]. However, it appears that virus may spread to distant sites within these cells, as similar appearing infected macrophages dominate in organs such as the brain [2, 3]. Attempts to mimic this infection in vitro have relied heavily on monocyte/macrophage cultures derived from PBMC rather than on actual peritoneal-type macrophages. Although monocyte cultures internalize FIPV much more efficiently than CRFK cells [4], virus replication in such cultures tends to be low and is not sustained in a chronic state as in nature. It is unlikely that the interaction between FIPV and macrophages can be easily mimicked by in vitro cell culture systems using other cell types.

The exact mechanism by which FIPV enters macrophages is unknown, although evidence suggests that it may not involve receptors used by other coronavirus species to infect intestinal or respiratory epithelium [5]. Several studies indicate that FIPV internalizes as immune complexes [6] through Fc receptors [7]. Indeed, antibodies to feline coronavirus (FECV or FIPV) enhance virus infection both in vitro [7] and in vivo [8]. The antibodies that mediate macrophage infection have been shown to be the same as those that inhibit FIPV infection in CRFK or Fcwf-4 cell in vitro and enhance the infectivity of FIPV in monocyte/macrophage cultures [9].

Apoptosis has been considered as a central feature of both experimentally-induced and naturally-occurring FIP [10, 11]. The emphasis of apoptotic events has been concentrated on lymphoid cells and not on infected macrophages. This bias is based on the common occurrence of lymphopenia in cats with FIP and the fact that macrophages appear largely unaffected in the face of infection. Moreover, apoptotic cells in lymphoid organs, when observed, are relatively scant and scattered [11].

When cats are experimentally infected with FIPV, whether they become immune or diseased is determined by how macrophages interact to replicating virus in the first 10–14 days and prior to the appearance of antibody [1]. Inhibition of virus replication with a protease inhibitor causes a rapid reversal of disease course and a return to normal in both experimental [12] and naturally occurring disease [13]. Therefore, it is apparent that the key to understanding FIP immunopathogenesis lies in how genes involved with immunity and inflammation are differentially expressed in FIPV infected macrophages during the earliest stage of infection.

The present study was an attempt to determine what happens to macrophages when they become persistently infected with FIPV and the host becomes diseased instead of immune. The tool used in this study was RNA-seq. To this end, this study compared the differential levels of mRNA expression in peritoneal cells from cats with experimentally induced wet FIP against normal peritoneal cells obtained by peritoneal lavage from naïve cats. The premise was that peritoneal cell populations would contain macrophages and that they would be the sole infected cell type. RNA-seq has been increasingly used to study changes in mRNA transcription in a number of virus infection models [14], starting with cell-lines infected in vitro [15] and then into laboratory animal models such as influenza A virus infection in mice [16]. Information gained from RNA-seq studies in animal models of infectious disease has opened the door to studies of natural infections of humans and other animal species.

## Materials and methods

### Sample collection

Cells from the peritoneal cavity were collected from four healthy cats by instilling 350–500 mL of warm lactated ringer’s solution into the peritoneal cavity, gently massaging the abdomen for 5–10 min, and then withdrawing as much fluid as possible by abdominal paracentesis. No prior stimulation was used to prevent non-specific activation. The total yield was 0.5–2.0 × 10^6^ cells per collection. Normal peritoneal cells consisted of an average of 14% eosinophils, 58% medium and large monocyte/macrophage type cells, 20% large and small lymphoid cells and 8% non-degenerate neutrophils based on morphology in Wright stained smears examined at 400× magnification.

**Figure 1:**
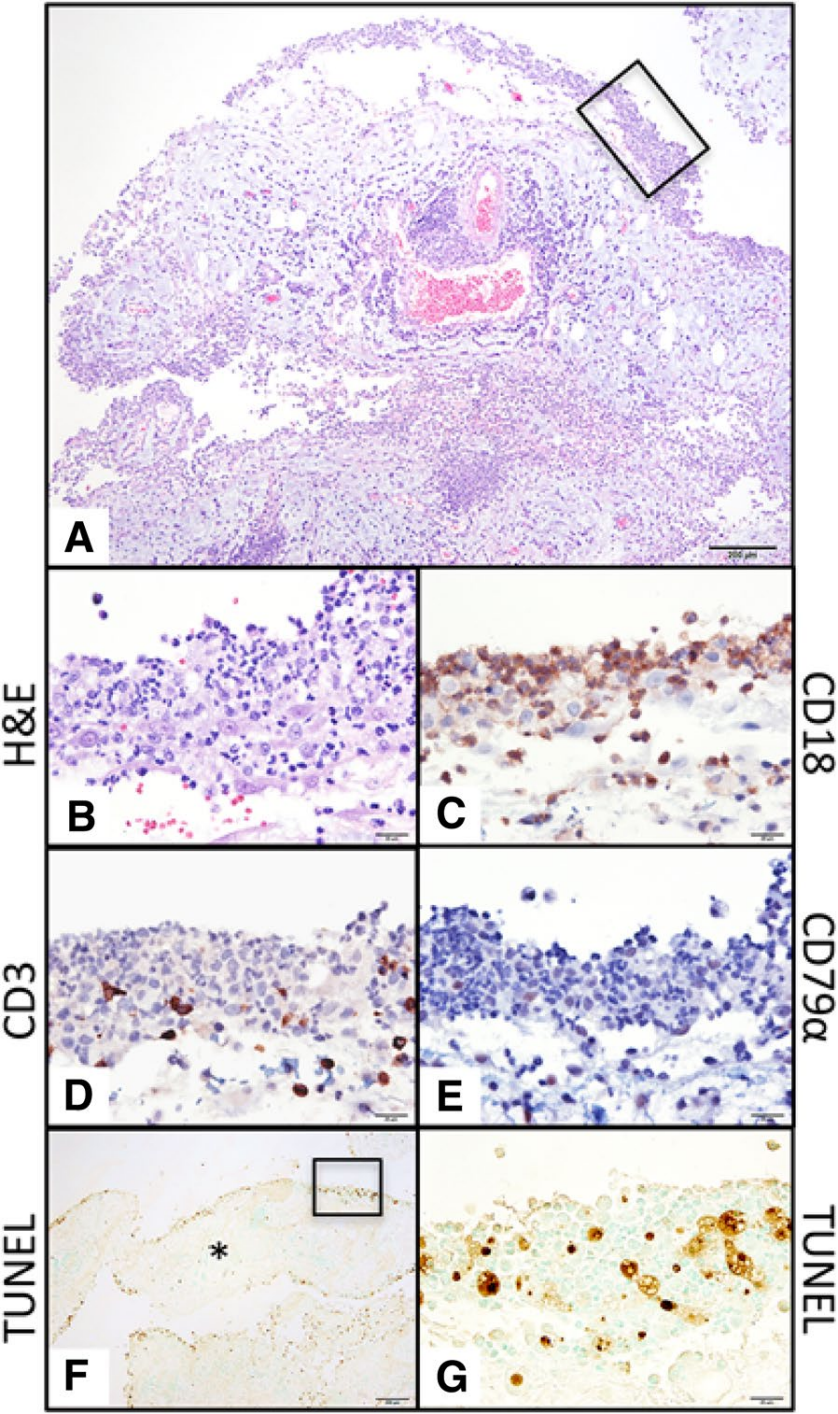
**Morphology, immunohistochemistry and TUNEL staining of peritoneal inflammatory cells of cats with experimentally-induced wet FIP. A** Omentum of an experimentally infected cat with FIPV is markedly expanded by inflammatory cells and covered with a thick sheet of peritoneal inflammation (rectangle area), ×40, hematoxylin and eosin stain. **B** ×400 magnification of the area outlined in **A** demonstrates sheets of inflammatory cells comprised predominantly of large vacuolated round cells (macrophages) and neutrophils. There are lesser numbers of scattered small lymphocytes, hematoxylin and eosin stain. **C** Most of the mononuclear cells stained positive for the CD18 cell surface marker, ×400, hematoxylin counterstain. **D** Staining for the CD3 T cell surface marker was limited to small numbers of small mononuclear cells, ×400, hematoxylin counterstain. **E** Staining for the CD79α B cell surface marker was limited to a low percentage of small mononuclear cells, ×400, hematoxylin counterstain. **F** Section of formalin-fixed, paraffin embedded and TUNEL stained inflamed omentum (*), ×40 magnification. Positive staining cells were concentrated in the inflamed surface. **G** High magnification of region outlined in the rectangle in **F**. TUNEL staining was pronounced in both cytoplasm and nuclei of large multi-vacuolated macrophages, while only a small proportion of more normal appearing monocyte/macrophages, lymphocytes and neutrophils stained positive in nuclei, ×400.

FIPV infected macrophages were obtained from ascites fluid of cats with experimentally-induced FIP that were part of other studies [1, 13]. Peritoneal cells from cats with FIP contained an average of 60% small to large macrophages, 10% small lymphoid cells, and 30% non-degenerate neutrophils based on morphology (Figure 1A). No eosinophils were observed. Light microscopic examination of chromogen immunohistochemistry revealed that most of the mononuclear cells stained strongly positive for CD18, a cell surface protein that is found at high concentration on monocyte/macrophages [17] (Figure 1B). A small proportion of small mononuclear cells stained positive for CD3 (T cells) (Figure 1C) or CD79a (B cells) (Figure 1D).

### RNA extraction

Total RNA was extracted from each sample using TRIzol^®^ reagent (Ambion: ThermoFisher, Waltham, MA, USA) and cleaned with RNA Clean and Concentrator-5™ (Zymoresearch, Irvine, CA, USA). After DNaseI (New England Biolabs, Ipswich, MA, USA) treatment, RNA was again cleaned with RNA Clean and Concentrator-5 and the quality of RNA was checked with an Agilent Bionalyzer 2100 using RNA nano chip (Agilent Technology, Santa Clara, CA, USA). RNA samples with RNA integrity numbers > 7 were subjected to library contraction.

### Library construction and deep sequencing

The construction of barcode indexed RNA-seq libraries and deep sequencing were performed by UC Davis genome center DNA core facility. In brief, poly-A RNA was enriched from 1 μg of total RNA with Kapa Stranded RNA-seq kit (KapaBiosystems, Cape Town, South Africa). The RNA-seq libraries were generated on a Sciclone NGS G3 liquid handler (Caliper Life Sciences, Alameda, CA, USA) and constructed libraries were analyzed with a Bioanalyzer 2100 (Agilent, Santa Clara, CA, USA) followed by the fluorometric quantification by Qubit (LifeTechnologies, Carlsbad, CA, USA). Libraries were pooled in equimolar ratios, quantified by qPCR with a Kapa Library Quant kit (KapaBiosystems), and sequenced on Illumina HiSeq 3000 platform (Illumina, San Diego, CA, USA) with paired-end 100 bp reads.

### Data processing and differential expression analysis

Data processing and differential expression analysis were done with Maverix WAVES™ RNA-seq 3.0 Analysis Kit. The quality of obtained raw sequencing data was checked using FastQC. Adapters and the portion of the reads with a quality score below 28 were trimmed using Trimmomatic [18]. Reads that were shorter than 20 bp after trimming were discarded. Remaining trimmed reads were mapped to the reference genome *Felis catus* 6.2 using STAR [19]. The abundance of annotated transcripts was estimated using Cufflinks [20] and reported as fragments per kilobase of exon per million fragments mapped (FPKM). Differential expression analysis was performed with DESeq [21] and only genes with a Log-2 Fold-Change (Log_2_FC) > 2.0 and < −2.0 were differentially expressed and significance assessed. Probability (*P*) values were adjusted according to the number of mRNAs tested (Bonferroni correction) and values < 0.05 considered to be significant.

There was an initial concern that capped RNA transcripts of FIPV RNAs present in infected peritoneal macrophages would obscure cellular mRNA. However, only a single RNA transcript was observed, and the number of transcripts was negligible compared to transcripts of host genes.

### Gene enrichment analysis

InnateDB is a publicly available database of the genes, proteins, and 18 780 experimentally-verified interactions and signaling pathways involved in the innate immune response of humans, mice and bovines to microbial infection [22]. The database captures an improved coverage of the innate immunity interactome by integrating known interactions and pathway databases including INOH, KEGG, PID BIOCARTA, NETPATH and READCROME. Pathways identified by InnateDB were further interrogated using KEGG [23] and QIAGEN gene lists.

### Histopathology and immunohistochemistry

Tissues obtained at necropsy from the previous experimental FIP study [1] were immediately placed in 10% buffered formalin and fixed for 24 h before being routinely trimmed and embedded into paraffin blocks. Tissues were examined histologically (hematoxylin and eosin stain), by immunohistochemistry (IHC), and TUNEL histochemistry. Immunohistochemistry was performed on 4 μm serial sections of formalin fixed and paraffin embedded tissues using a streptavidin biotin detection system (Biocare Medical, Concord, CA, USA). Mouse monoclonal antibodies to FIPV (Custom Monoclonal International, clone FIPV3-70, 1:200), CD18 (Peter Moore, UC Davis, clone Fe3.9F2), CD3 (Peter Moore, UC Davis, clone CD3-12), and CD79a (Dako Inc., Carpinteria, California, clone HM57) were used for IHC. Before applying the primary antibodies, slides were steam pretreated in citrate buffer (Dako S1699) at 98 °C for 20 min followed by a 20-min cooling. They were then washed in PBS and blocked with 10% normal horse serum for 20 min. Amino ethyl carbazole (AEC, Dako Corp.) was used as the chromogen. Sections were counterstained with Mayer’s hematoxylin. Substituting a matched mouse IgG correlate for the primary antibody served as the negative control.

Direct immunofluorescent antibody (DIFA) staining was done on frozen sections. The globulin fraction of high feline coronavirus tittered serum was obtained from cats with FIP and conjugated with fluorescein isothiocyanate [8]. Tissue specimens with FIP lesions (e.g., omentum) were blocked in O.C.T. medium (Tissue-Tek^®^, Sakura Finetek, USA, Inc) and snap-frozen in isopentane and liquid nitrogen. Sections were cut on a cryostat-microtome and fixed with acetone in preparation for DIFA staining.

### In situ TUNEL assay

TUNEL staining was performed using in situ Apoptosis Detection Kit according to the manufacturer’s instruction (Abcam, Cambridge, UK). Formalin-fixed paraffin embedded sections from diseased tissues were permeabilized with protease K, and labeled with HRP-conjugated dUTP. Labeled cells were visualized with DAB colorimetric detection.

## Results

### Read quality and the result of differential expression analysis

More than 70 million reads were obtained for each sample group after trimming of low quality reads. The retained reads were mapped to the reference sequence and 45–46% of reads were mapped (Table 1). Differential expression analysis identified 5621 genes as deregulated in cells from peritoneal exudates of cats with experimentally-induced FIP. Among those genes, 956 showed equal or more than 2 Log Fold Change (Log_2_FC) and 1589 genes showed ≤ −2 Log_2_FC.

**Table 1:** **Number of samples tested, average read numbers, and percentage of reads mapped to reference sequences**

Sample	Sample numbers	Average read numbers (after trimming)	Average number of reads mapped to reference sequence	Average % reads mapped
FIP peritoneal exudate cells	11	74 948 182	34 292 273	45.75
Control peritoneal cells	4	75 560 000	34 826 250	46.09

### Pathways identified by InnateDB

Eighteen pathways were up-regulated in peritoneal cells harvested from the ascites fluid of cats with experimentally-induced FIP by InnateDB analysis at a significance of *P*_adjusted_ < 0.05 (Table 2). The up-regulated pathways were associated with responses to infectious agents (Toll- and Rig-I-like receptor signaling, cytokine–cytokine receptor interaction, apoptosis, cytosolic DNA-sensing, Jak-STAT signaling, NK cell mediated cytotoxicity and NOD-like receptor signaling); specific infectious diseases (hepatitis C, leishmaniasis, American and African trypanosomiasis, toxoplasmosis); allograft rejection and graft-versus host disease; and autoimmune disorders including type I diabetes mellitus and autoimmune thyroiditis. However, the osteoclast differentiation pathway did not fit the pattern of the other upregulated pathways. Three pathways were significantly downregulated, all involving cellular functions (Table 2).

**Table 2:** **Description of 18 pathways in InnateDB that were significantly (** ***P*** **< 0.05) up-regulated and 3 pathways significantly downregulated in peritoneal exudate cells from cats with FIP compared to peritoneal cells from healthy cats**

Pathway name	Genes in InnateDB	Dysregulated gene count	*P*_adjusted_
InnateDB pathways that were significantly upregulated			
Toll-like receptor signaling pathway	67	28	1.47E−06
RIG-I-like receptor signaling pathway	42	21	2.23E−06
Cytokine–cytokine receptor interaction	148	45	5.97E−06
Apoptosis	60	24	3.26E−05
Cytosolic DNA-sensing pathway	34	17	3.97E−05
Graft-versus-host disease	13	10	4.33E−05
Hepatitis C	80	27	2.48E−04
Leishmaniasis	41	17	6.61E−04
Allograft rejection	14	9	9.82E−04
Type I diabetes mellitus	18	10	0.00170
Jak-STAT signaling pathway	90	26	0.00404
Chagas disease (American trypanosomiasis)	72	22	0.00539
African trypanosomiasis	25	11	0.00712
Osteoclast differentiation	80	23	0.00838
Natural killer cell mediated cytotoxicity	66	20	0.00867
Autoimmune thyroid disease	15	8	0.00896
Toxoplasmosis	78	21	0.02566
NOD-like receptor signaling pathway	36	12	0.03707
InnateDB pathways that were significantly downregulated			
DNA replication	29	18	5.21E−04
Cell cycle	82	34	0.00446
Basal cell carcinoma	32	17	0.00808

### Differential expression of genes associated with M1 (immune) and M2 (phagocytic) macrophage differentiation

Eighteen of 23 genes associated with M1 macrophage polarization were detected by RNA-seq in peritoneal cells from FIPV infected versus normal cats and 13 of them were significantly over-expressed and no gene was under-expressed (Table 3). In comparison, only three detected genes in the M2 pathway were significantly deregulated; two were significantly up-regulated and one was significantly down-regulated (Table 4). JAK2 was significantly upregulated and shared by both M1 and M2 polarization pathways.

**Table 3:** **Differential expression profile of genes involved in M1 macrophage polarization in peritoneal exudate cells from cats with FIP compared to peritoneal cells from healthy cats**

Gene symbol	Description	Log_2_FC	*P*_adjusted_
*IL6*	*Interleukin 6*	*4.86971*	*0.0006*
*NOS2*	*Nitric oxide synthase 2, inducible*	*4.21906*	*2.16E−05*
*NFKB2*	*Nuclear factor of kappa light polypeptide gene enhancer in B-cells 2 (p49/p100)*	*4.14805*	*5.83E−16*
*IL12B*	*Interleukin 12B*	*3.83085*	*2.91E−06*
*RELB*	*v-rel avian reticuloendotheliosis viral oncogene homolog B*	*3.73979*	*2.65E−16*
*STAT1*	*Signal transducer and activator of transcription 1, 91* *kDa*	*2.58559*	*2.72E−11*
*STAT2*	*Signal transducer and activator of transcription 2, 113* *kDa*	*2.25218*	*5.60E−09*
*JAK2*	*Janus kinase 2*	*2.00000*	*4.96E−07*
*TLR4*	*Toll-like receptor 4*	*1.90593* ^a^	*8.94E−07*
*NFKB1*	Nuclear factor of kappa light polypeptide gene enhancer in B-cells 1	1.70639	1.07E**−**05
*RELA*	v-rel avian reticuloendotheliosis viral oncogene homolog A	1.48184	0.00019
*CIITA*	Class II, major histocompatibility complex, transactivator	1.41143	0.00014
*CSF2RB*	Colony stimulating factor 2 receptor, beta, low-affinity (granulocyte–macrophage)	1.36877	0.0252
*IRF3*	Interferon regulatory factor 3	0.99868	0.01143
*MAPK14*	Mitogen-activated protein kinase 14	0.51622	0.27282
*IFNGR1*	IFN-g receptor 1	0.46379	0.39809
*IRF5*	Interferon regulatory factor 5	0.23661	0.69315

Genes listed in italics print were significantly up-regulated. No genes were significantly down-regulated.

The list was adapted from a previous publication [70].

^a^Slightly less than Log_2_FC cutoff but *P* value highly significant.

**Table 4:** **Differential expression profile of genes involved in M2 macrophage polarization in peritoneal exudate cells from cats with FIP compared to peritoneal cells from healthy cats**

Gene symbol	Description	Log_2_FC	*P*_adjusted_
*IL4R*	*Interleukin 4 receptor*	*2.8196*	*7.44E−13*
*JAK2*	*Janus kinase 2*	*2*	*4.96E−07*
*STAT6*	Interferon regulatory factor 3	0.99868	0.01143
*JAK3*	Janus kinase 3	0.949332	0.01951
*IL13RA1*	Interleukin 13 receptor, alpha 1	0.29314	0.67891
*CSF1R*	Colony stimulating factor 1 receptor	−0.67799	0.05583
*IL10*	Interleukin 10	−1.13254	0.02941
*MRC1*	Mannose receptor, C type 1	−1.22721	0.00145
*IL13RA2*	Interleukin 13 receptor, alpha 2	−1.66057	0.17644
PPARG	Peroxisome proliferator-activator	−3.45082	9.70E −08

Genes listed in italics were significantly up-regulated and underlined genes significantly down-regulated.

### Upregulation of pro-apoptotic genes

InnateDB identified apoptosis as the fourth most significantly upregulated pathway based on differential gene expression. Table 5 lists individual genes in the QIAGEN human apoptosis PCR known to positively regulate programmed cell death.

**Table 5:** **Differential expression profile of genes that positively regulate apoptosis in peritoneal exudate cells from cats with FIP compared to peritoneal cells from healthy cats. The list was modified from QIAGEN human apoptosis PCR array**

Gene symbol	Description	Log_2_FC	*P*_adjusted_
*FAS*	*Fas cell surface death receptor*	*4.44221*	*1.66E−19*
*CD70*	*CD70 molecule*	*3.91193*	*0.0008*
*TRAF2*	*TNF receptor-associated factor 2*	*3.69038*	*2.77E−19*
*FASLG*	*Fas ligand (TNF superfamily, member 6)*	*3.51023*	*0.00019*
*BAK1*	*BCL2-antagonist/killer 1*	*3.41627*	*1.85E−17*
*TNF*	*Tumor necrosis factor*	*3.15917*	*2.35E−15*
*TNFSF10*	*Tumor necrosis factor (ligand) superfamily, member 10*	*3.10015*	*5.33E−15*
*BID*	*BH3 interacting domain death agonist*	*2.46508*	*1.95E−09*
*CD27*	*CD27 molecule*	*2.30807*	*0.01807*
*BCL2L11*	*BCL2-like 11 (apoptosis facilitator)*	*2.25067*	*6.41E−06*
*CASP10*	*Caspase 10, apoptosis-related cysteine peptidase*	*2.12608*	*3.21E−08*
*CASP8*	*Caspase 8, apoptosis-related cysteine peptidase*	*2.00000*	*3.56E−07*
*BCL10*	B-cell CLL/lymphoma 10	1.49878	0.00021
*CIDEA*	Cell death-inducing DFFA-like effector a	1.45359	0.26372
*TNFSF9*	Tumor necrosis factor (ligand) superfamily, member 9	1.33377	0.00087
*BRAF*	B-Raf proto-oncogene, serine/threonine kinase	1.27788	0.00326
*DFFA*	DNA fragmentation factor, 45 kDa, alpha polypeptide	0.92582	0.01018
*BNIP3L*	BCL2/adenovirus E1B 19 kDa interacting protein 3-like	0.84923	0.04892
*BAX*	BCL2-associated X protein	0.77062	0.05153
*BIK*	BCL2-interacting killer (apoptosis-inducing)	0.66673	0.28001
*TRADD*	TNFRSF1A-associated via death domain	−0.0058	0.96717
*ABL1*	ABL proto-oncogene 1, non-receptor tyrosine kinase	−0.49077	0.19821
*TP73*	Tumor protein p73	−0.76667	0.17226
*TP53BP2*	Tumor protein p53 binding protein 2	− 0.76713	0.04169
*TP53*	Tumor protein p53	−0.99445	0.005241
*CASP2*	Caspase 2, apoptosis-related cysteine peptidase	−1.18226	0.0037
*DAPK1*	Death-associated protein kinase 1	−1.59291	3.29E−06
*NOD1*	Nucleotide-binding oligomerization domain containing 1	−1.61573	3.08E−06
TNFSF8	Tumor necrosis factor superfamily member 8	−4.26451	7.28E−24

Genes in italics were significantly up-regulated  and underlined genes significantly down-regulated.

### Histopathology, immunohistochemistry and the identification of major cellular targets for apoptosis

Severe serositis was observed in the abdominal cavity of cats with FIP. The serosal surfaces of the abdominal organs including the liver, spleen, kidney and intestines were expanded on histology by pyogranulomatous inflammation, fibrin, and edema. The omentum and mesentery were diffusely irregularly thickened by sheets of inflammatory cells on the peritoneal surface and multifocally within the adipose parenchyma. The sheet of inflammatory cells on the peritoneal surface of the omentum was comprised of large numbers of macrophages and neutrophils, and lesser numbers of scattered small lymphocytes (Figures 1A and B). The adipose tissue of the omentum was multifocally expanded by lymphoid aggregates centered around blood vessels and scattered lymphocytes, macrophages and neutrophils. Immunohistochemistry demonstrated that a large proportion of cells in the exudate lining the omentum were large CD18 immunoreactive round cells (Figure 1C), consistent with monocyte/macrophages. A small proportion of scattered cells were immunoreactive for CD3 (T lymphocyte marker, Figure 1D) and very few cells immunoreactive for CD79α (B lymphocyte marker, Figure 1E).

**Figure 2:**
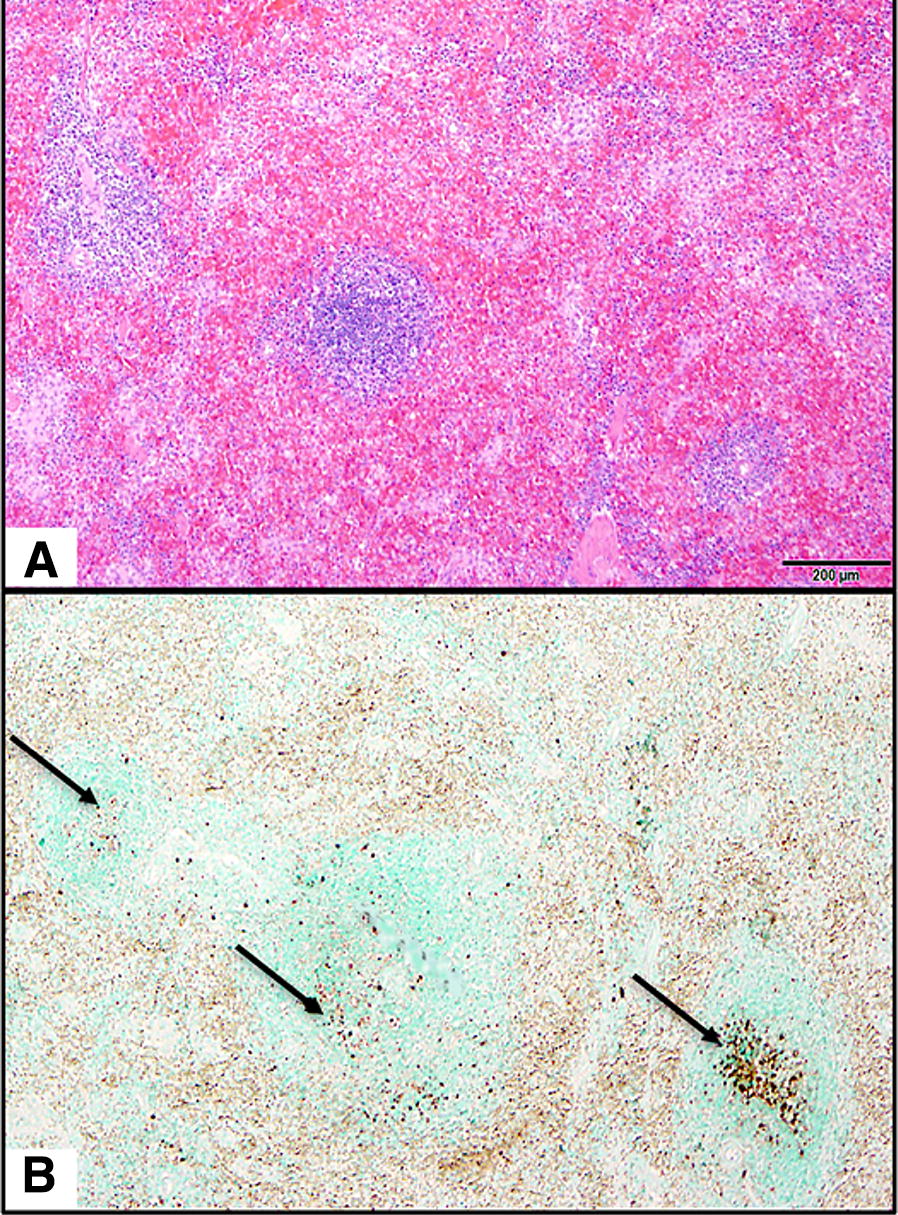
**TUNEL stained section of spleen from a cat with FIP. A** Section from spleen of a cat with FIP containing three periarteriolar lymphoid sheaths, ×40 magnification. **B** Strongly TUNEL positive cells are seen in varying proportions in the central B cell zones of lymphoid follicles (arrows) and among sparse lymphocyte appearing cells scattered among the sinusoids and greenish stained marginal zones. Staining was mainly within nuclei and not cytoplasmic, ×40 magnification.

The specific cellular targets for apoptosis were determined by TUNEL staining and the FIPV infection status of stained cells interrogated by a combination of DIFA staining using polyclonal cat Ig and indirect immunoperoxidase staining using a mouse monoclonal antibody to the FIPV N protein. Omentum with FIP lesions was used as the target tissue. TUNEL-positive cells were scattered on the peritoneal surface of the inflamed omentum and comprised ~ 10% of the total cells (Figure 1F). The morphology of the strongly staining cells was characteristic for activated macrophages, i.e., they were large and highly vacuolated (Figure 1G). Apoptosis in the omentum was limited to large highly vacuolated macrophages and TUNEL stained lymphocytes and neutrophils were sparse even though there was an extensive lymphocytic/neutrophilic infiltrate. Therefore, the spleen was also examined by IHC and TUNEL staining. Unlike the omentum, lymphoid cells were arranged in a more orchestrated (reactive) manner around existing structures such as germinal centers and marginal zones. Apoptotic cells were concentrated in the germinal centers of splenic lymphoid follicles (Figures 2A and B). These were presumably B cells based on IHC. This response may have been related or unrelated to the FIPV infection. Some unidentified cells in the marginal zones were faintly stained by TUNEL.

**Figure 3:**
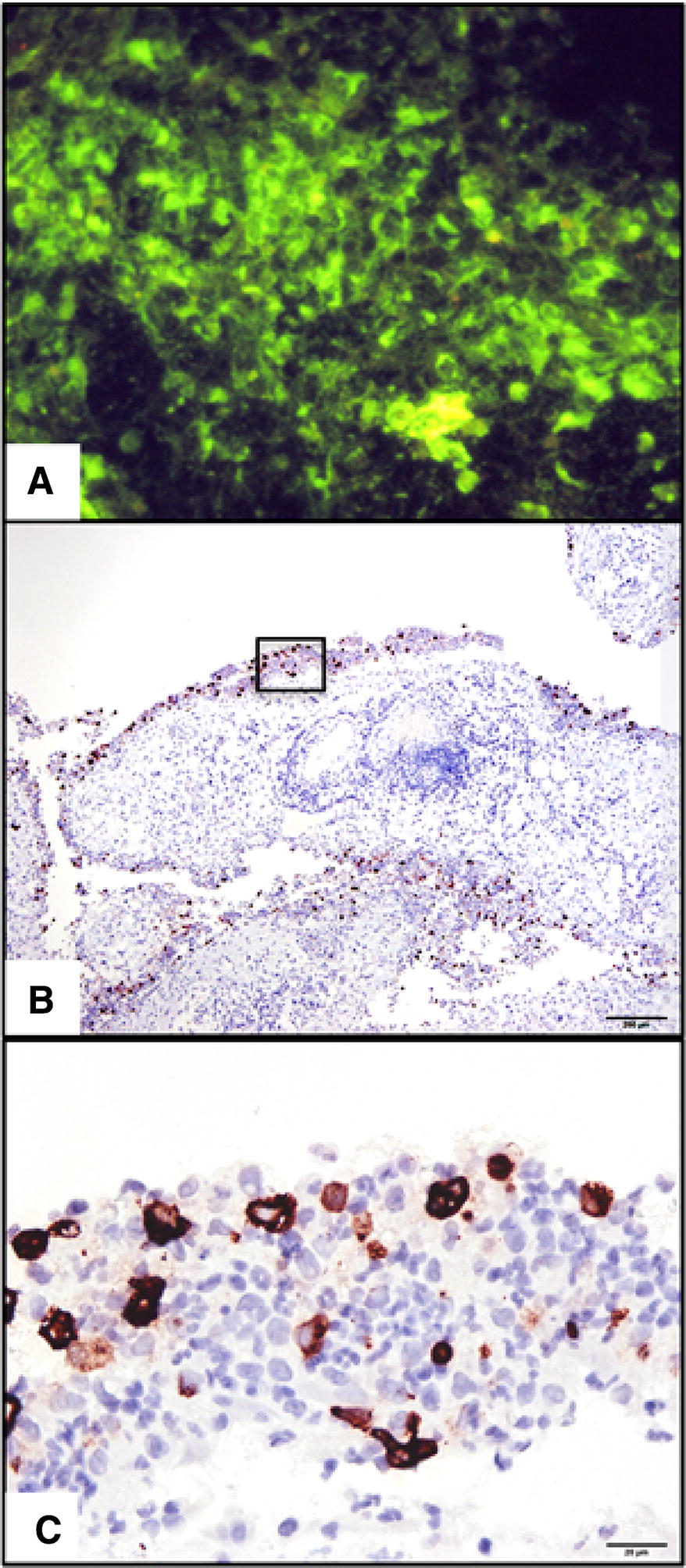
**Identification of FIPV antigens in inflamed omentum from a cat with experimentally-induced FIP. A** OCT embedded and cryostat sectioned omentum from cat with experimentally-induced effusive FIP stained by DIFA using cat globulin from FIPV infected cats. Intense fluorescence was seen in most monocyte/macrophage appearing cells within a pyogranuloma, ×400, Evan’s blue counterstain. **B** A formalin-fixed and paraffin embedded section from the identical diseased region of omentum as shown in **A**. The section was reacted with mouse monoclonal antibody to the N protein of FIPV followed by goat anti-mouse Ig conjugated to horse radish peroxidase, ×40, hematoxylin counterstain. **C** High power view of region outlined in **B**. Staining for FIPV N protein was concentrated in cytoplasm of large multi-vacuolated macrophages with faint staining in smaller monocyte/macrophage appearing cells, ×400, hematoxylin counterstain.

The polyclonal cat globulin produced intense cytoplasmic staining in virtually all the small to large macrophage-type cells found as focal aggregates in the omentum (Figure 3A), while the mouse monoclonal antibody to the N protein intensely stained primarily the large highly vacuolated macrophages (Figures 3B and C). The differential intensity of staining with the two procedures indicated that almost all macrophage-type cells contain viral antigens, while the greatest concentration of assembling virions was in the largest multi-vacuolated cells.

### Differential expression of MHC-I and -II receptor mRNAs on FIPV infected peritoneal macrophages

The MHC of the cat is known as the feline leukocyte antigen (FLA), the dog MHC as the dog leukocyte antigen (DLA), and the human MHC as the HLA (human leukocyte antigen). The mRNA for the MHC class I FLAI-K-feline receptor was the only transcript that was reported as significantly dysregulated, although not quite at the assigned ≥ Log_2_FC cutoff. RNAs for the MHC class I and II human and canine orthologues were not dysregulated (Table 6).

**Table 6:** **Differential expression profile of FLA, HLA and DLA orthologous receptor RNAs in peritoneal exudate cells from cats with FIP compared to peritoneal cells from healthy cats**

Gene symbol	Description	Log_2_FC	*P*_adjusted_
*FLAI-K-feline*	*Feline class I antigen-alpha chain-like class I antigen*	*1.7066*	4.95E−06*
*LOC101084108*	HLA class II histocompatibility antigen, DM alpha chain	0.24295	0.50201
*LOC101082906*	HLA class II histocompatibility antigen DO beta chain	0.06259	0.8714
*LOC101090265*	HLA class II histocompatibility antigen, DM beta chain	−0.52204	0.24494
*LOC101098301*	DLA class II histocompatibility antigen, DR-1 beta chain	−0.61283	0.2607
*LOC101088734*	HLA class II histocompatibility antigen DR alpha chain-like	−0.63486	0.24216
*HMHA1*	Minor human histocompatibility protein HA-1	−0.67421	0.0512

Genes listed in italics print were significantly (*P* < 0.05) up-regulated.

*Log_2_FC  slightly below 2.0 but *P* value highly significant.

### Expression of Fc receptor genes

Table 7 lists the known alternative (Fc) receptors. All of them except for *FcεRII* are found on macrophage/monocytes. FcγRIIIA mRNA was the only Fc receptor mRNA that was significantly upregulated. Feline orthologues for several Fc receptor genes were not detected (ND).

**Table 7:** **Differential expression profile of alternative Fc receptors in peritoneal exudate cells from cats with FIP compared to peritoneal cells from healthy cats**

Gene	Ligand	Cell distribution	Effect following binding to antibody	Log_2_FC	*P*_adjusted_
*FcγRIIA (CD32)*	IgG	Macrophages neutrophils eosinophils, DC, platelet	Phagocytosis, degranulation eosinophils	ND	
*FcγRIIB1 (CD32)*	IgG	B cells mast cells	No effect on phagocytosis, inhibition of cell activity	1.03	0.011
*FcγRIIIA (CD16a)*	*IgG*	*B-cell, NK cells, neutrophils, macrophages, FDC*	*Induction of ADCC, cytokine release by macrophage*	*2.32*	*8.20E−10*
*FcγRIIIB (CD16b)*	IgG	Eosinophils macrophages neutrophils mast cells follicular dendritic cells	Induction microbe killing	ND	
*FcεRI*	IgE	Mast cells, monocytes, eosinophils basophils langerhans cells	Degranulation eosinophils, phagocytosis	ND	
*FcεRII (CD23)*	IgE	B cells, eosinophils. Langerhan cells	Allergic sensitization, IgE transport	0.93	0.237
*FcαRI (CD89)*	IgA	Monocytes macrophages neutrophils, eosinophils	Phagocytosis, microbe killing	ND	
*Fcα/μR*	IgA, IgM	B cells, mesangial cells, macrophages	Endocytosis, induction microbe killing	0.88	0.44
*FcRn (FCGRT)*	IgG	Monocytes macrophages dendritic cells epithelial cells endothelial cells	Maternal IgG transfer placenta and milk, protects IgG from degradation	−0.54	0.200

Genes in italics were significantly up-regulated. Several RNAs were not detected (ND).

### Coronavirus-associated attachment receptors

DC-SIGN (CD209) showed significant down-regulation, while known receptor molecules for other coronavirus species of people and animals were either marginally down-regulated (DDP4) or their RNAs not detected (ND) (L-Sign, ACE2, APN) (Table 8).

**Table 8:** **Differential expression profile of coronavirus-associated receptors**

Gene symbol	Description	Log_2_FC	*P*_adjusted_
DC-SIGN	CD209 molecule	−3.15568	2.00E−11
L-SIGN	CD209 ligand	ND	0.00379
DDP4	Dipeptidyl peptidase 4	1.5332	
ACE2	Angiotensin converting enzyme 2	ND	
APN	Aminopeptidase N	ND	

Underlined genes were significantly down-regulated in peritoneal exudate cells from cats with FIP.

### Differential expression profile of genes related to Th1 and Th2 cell differentiation pathways

The failure of FIPV infected cats to mount an effective initial immune response has been attributed to the inhibition of Th1 (cell-mediated) and enhancement of Th2 (antibody) immune responses. Therefore, the expectation is that Th2 polarization would dominate in these cats, but it appeared at firsthand that the Th1 pathway was dominant (Table 9). However, the KEGG Th2 pathway did not list PRDMI, IL6 and BAFF among important B cell related genes. When these were added to the list, upregulation of Th2 polarization became more evident (Table 9).

**Table 9:** **Differential expression profile of genes related to Th1 and Th2 cell differentiation pathways in peritoneal exudate cells from cats with FIP compared to peritoneal cells from healthy cats**

Gene symbol	Description	Log_2_FC	*P*_adjusted_
KEGG Th1 polarization			
*IFNG*	*Interferon, gamma*	*6.02974*	*7.48E−10*
*IL12B*	*Interleukin 12B*	*3.83086*	*2.91E−06*
*IL12RB1*	*Interleukin 12 receptor, beta 1*	*3.82454*	*2.62E−20*
*IL12RB2*	*Interleukin 12 receptor, beta 2*	*3.22364*	*3.50E−08*
*STAT1*	*Signal transducer and activator of transcription 1, 91* *kDa*	*2.58559*	*2.72E−11*
*TYK2*	*Tyrosine kinase 2*	*2.52411*	*8.23E−09*
*TBX21*	*T-box 21*	*2.49393*	*0.00033*
JAK2	Janus kinase 2	1.93023	4.96E**−**07
MAML1	Mastermind-like 1 (Drosophila)	1.82799	4.14E**−**06
*RBPJL*	Recombination signal binding protein for immunoglobulin kappa J region-like	1.67996	0.22008
*RELA*	v-rel avian reticuloendotheliosis viral	1.48184	0.00019
*STAT4*	Signal transducer and activator of transcription 4	1.46177	0.00289
*IFNGR1*	Interferon gamma receptor 1	0.46379	0.39809
DLL1	Delta-like 1 (Drosophila)	-2.74765	1.65E −10
NOTCH3	Notch 3	-4.00499	4.20E −15
KEGG Th2 polarization			
*IL6*^**a**^	*Interleukin 6*	*4.86971*	*0.00071*
*IL2RG*	*Interleukin 2 receptor, gamma*	*4.52368*	*7.33E−20*
*PRDMI*^**a**^	*Blimp-1, PR domain containing 1, with ZNF domain*	*3.77121*	*5.13E−10*
*CD40*^**a**^	*TNFSF5, tumor necrosis factor super family 5*	*3.36350*	*1.65E−17*
*IL4R*	*Interleukin 4 receptor*	*2.8196*	*7.44E−13*
*BAFF*^**a**^	*TNFS13B, tumor necrosis factor super family 13B*	*1.7417*	*4.41E−06* ^b^
*GATA3*	GATA binding protein 3	1.7299	0.01676
*RBPJL*	Recombination signal binding protein for immunoglobulin kappa J region-like	1.6799	0.22008
*IL2RA*	Interleukin 2 receptor, alpha	0.3470	0.67473
*STAT6*	Signal transducer and activator of transcription 6, interleukin-4 induced	0.1629	0.75754
*IL4*	Interleukin 4	0	0.01694
*NOTCH2*	Notch 2	0.1502	0.58684
*MAML3*	Mastermind-like 3 (Drosophila)	0.7689	0.02175
JAG2	Jagged 2	−2.34773	6.33E −10

Table was produced based on KEGG Th1 and Th2 cell differentiation pathway (KEGG map04658). Genes in italics were significantly up-regulated and Underlined genes were significantly down-regulated (*P* < 0.05).

^a^Genes added to KEGG Th2 cell polarization pathway based on FIP literature.

^b^ Slightly below > 2.0 Log_2_FC but *P* value highly significant.

## Discussion

### Summary of gene expression and potential limitations of RNA-seq

Two related studies have described differential gene expression in normal and FIPV infected cell cultures [24, 25]. They identified 18 899 of 19 046 annotated genes; a total of 61 genes were differentially expressed, 44 up-regulated and 17 down-regulated. Most of the genes were those associated with monocytes-macrophages, Th1 cell functions, and apoptosis, mirroring the present findings. Although the results of the present study supported these earlier published results, such studies used serotype II, tissue-culture adapted FIPV on an atypical host cell (e.g., CRFK cells) and employed a limited number of replicates. The present study used RNA-seq to compare gene expression in peritoneal cells from four healthy cats with gene expression from peritoneal cells of 11 cats that had been experimentally infected with non-tissue culture adapted serotype I FIPV, which more closely paralleled the natural infection. The present study also identified a greater number of significantly dysregulated genes (5621), with many of them expressed at levels 200-fold higher (956) or lower (1589) in FIPV infected versus normal peritoneal cells.

There were two inherent limitations of the present study, purity of cells and accuracy of RNA-seq as a predictor of cytokine translation. Peritoneal cells were not purified, because any purification method would likely cause significant changes in differential gene expression profiles. This lack of purity was not seen as a problem, as peritoneal cells collected from both healthy and diseased cats contained a predominance of macrophage-type cells. Eosinophils were present in the normal peritoneal exudate cell population but made no contribution whatsoever to the cells in peritoneal exudate from cats with FIP. Neutrophils were present in both populations but did not appear grossly activated in either situation in stained smears. Finally, the differentially expressed genes were associated mainly with macrophage type cells and not with neutrophils. Therefore, the assumption was that the present differential gene expression profiles reflected primarily macrophage-type cells.

The second limitation is more important, because it is presumed that there is only about 40% agreement between differentially expressed mRNA and mRNA-protein levels [26]. Therefore, some researchers believe that mRNA levels cannot be used as surrogates for corresponding protein levels without verification. Despite the inconsistencies, researchers still see value in RNA-seq analyses, especially when translated proteins have a short half-life and the genetic complexity of the system being studied is low [27]. It appears that the half-life of cytokines generated by infected/activated macrophages in this experimental FIPV infection model is very short. Fever and lymphopenia appear suddenly with the onset of peritonitis [1] and disappear within hours of virus inhibition using a potent anti-viral drug [12]. However, the magnitude and complexity of cytokine responses observed in the present study appears high rather than low.

### Deregulated gene expression in peritoneal exudate cells from cats with FIP compared to peritoneal cells from healthy cats

Many individual genes were dysregulated in peritoneal cells from FIPV infected cats, which was not surprising given its infectious origin and intense inflammatory responses. A more focused interpretation of the data was obtained from functional gene pathways, especially those identified by InnateDB, Qiagen, and KEGG databases. Only three InnateDB pathways were significantly under-expressed, all related to normal cellular functions, while 18 pathways were over-expressed. Four of the most significant up-regulated pathways were associated with microbe recognition—toll-like receptor signaling, RIG-I-like receptor signaling, cytosolic DNA-sensing and NOD-like receptor signaling pathways. Recognition of a foreign invasion, and in this case by an RNA virus, is the essential first step in the initiation of an immune and/or inflammatory response. It is also noteworthy that five of the eighteen most significantly upregulated InnateDB pathways were associated with infectious agents such as hepatitis C virus (another RNA virus), leishmaniasis, American and African trypanosomiasis, and toxoplasmosis. These diseases are all associated with pathogens of macrophages (except for hepatitis C virus) that result in persistent intracellular infections and disease. FIPV is the only RNA virus known to cause granulomatous lesions that mirror those of classic non-viral macrophage pathogens [28, 29] and it is understandable why systemic feline mycobacteriosis is nearest in clinical and pathologic features to FIP [30]. It is also noteworthy that two InnateDB pathways resembled those associated with graft versus host disease and allograft rejection, while two other pathways were shared with type I diabetes mellitus and autoimmune thyroiditis. These conditions all involved problems in differentiating self from non-self that lead to inflammation. Reasons for upregulation of the osteoclast differentiation pathway was more difficult to understand.

### Differential expression of genes associated with M1 (immune) and M2 (phagocytic) macrophage differentiation

The present study indicated that macrophages in ascites from experimentally FIPV infected cats were of the M1 (inflammatory, immune) type. Many mRNAs associated with M1 activation were significantly over-expressed in the peritoneal exudate cells, while mRNAs associated with M2 activation were either down-regulated or non-detectable. M1 (killer) macrophages are activated by LPS and IFN-gamma, and secrete high levels of IL12 and low levels of IL10. This pattern was confirmed by mRNA expression with IL10 mRNA being down-regulated and IL12 mRNA upregulated. Although brief expression of mRNAs associated with M1 macrophages may be beneficial, excessive and/or prolonged M1 polarization can lead to pathogen-induced inflammation rather than immunity [31]. This appears to be the case in the infected cats in the present study.

### Deregulated genes in apoptosis signaling pathway

The most conspicuous target for apoptosis was the large virus laden multi-vacuolated macrophages present in lesions and effusions. The staining pattern in the large multi-vacuolated macrophages was more cytoplasmic, while TUNEL staining of lymphocytes and neutrophils resulted in a classical nuclear staining. This differential cytoplasmic TUNEL staining-pattern has been previously described in passing in one other study [11]. The specific and unique pattern of TUNEL stain in these cells suggested that apoptosis is modulated in a different manner in this cell type. A novel caspase-independent pathway has been shown to trigger the cell death pathway in macrophages with high numbers of intracellular *M. tuberculosis* bacilli [32].

Staining for FIPV in this study was done with both mouse monoclonal and feline polyclonal antibodies. The monoclonal antibody was specific for the nucleocapsid (N) protein, a protein that plays a central role in focusing viral transcription and assembly [33] in large membrane bound replication-transcription complexes [34]. Therefore, it was not surprising that staining for N protein was concentrated in the large multi-vacuolated cells. In contrast, polyclonal antibody used with DIFA demonstrated intracellular viral proteins in macrophages with various morphology, which suggesting they are at different stages of differentiation and infection. A relationship between cell death and high levels of intracellular *Mycobacteria tuberculosis* has also been described [32]. In this scenario, more immature and less virus-laden peritoneal monocyte/macrophages fail to undergo programmed cell death, while older virus laden cells are specifically targeted for destruction by a mechanism distinct from classic apoptosis [35]. Such a process assures persistence of infected cells, while allowing for efficient dissemination of the pathogen [36].

### Differential expression of MHC-I and -II receptor mRNAs by FIPV infected peritoneal cells

Macrophages play a key role in initiating Th1 and Th2 immune responses using specific MHC receptors that present self and foreign peptides from within the cell to CD8+ (cellular immunity) or CD4+ (humoral immunity) T cells. Only FLAI-K, the feline MHC class I receptor protein, was noticeably up-regulated among mRNAs in cats with FIP. MHC I molecules have been detected on the cell surfaces of infected macrophages from cats with naturally-occurring FIP [37], indicating that the FLAI-K proteins were also produced in the present cats. The function of FLAI-K is to present immunogenic FIPV peptides to CD8+ T cells, which might be expected to result ultimately in the destruction of infected monocyte/macrophages. This poses a paradox, because most of the monocyte/macrophages from cats in this study were not apoptotic. However, viral antigens have not been detected on the membranes of macrophages from cats with naturally-occurring FIP [37]. This observation may be significant as many viruses have evolved mechanisms to interfere with antigen presentation to MHC I molecules as a strategy to evade the immune system [38].

The lack of upregulation of MHC class II receptor genes in peritoneal cells from cats with FIP may be of some significance. MHC class II molecules enhance TLR mediated innate immune responses and a failure to increase MHC class II molecules may have a suppressing effect on innate immune responses [39].

### Differential expression of known coronavirus attachment receptors

The receptor molecules used by various coronavirus species are known [40]. Among alphacoronaviruses, human bronchitis virus NL63 uses ACE2, HCOV-229E L-SIGN, and TGEV and PRCV bind to aminopeptidase N (APN). The betacoronaviruses MERS and SARS bind to dipeptidyl peptidase-4 (DPP4) and angiotensin-converting enzyme 2 (ACE2), respectively. However, the route by which FIPV infects monocytes and macrophages is much less certain, but both APN and DC-SIGN have been implicated at one time or another. Aminopeptidase N was initially identified as the receptor for all group I feline, canine, porcine and human coronaviruses [41] and later cited as a receptor for serotype II but not for serotype I FIPVs [42]. Subsequent in vitro studies also indicated that the predominant serotype I strains of FIPVs found in nature do not use aminopeptidase N as a receptor [5, 43, 44]. Blocking of aminopeptidase N receptors has also no effect on serotype I FIPV binding and infection of feline monocyte cultures [45]. mRNA for aminopeptidase N was not identified in peritoneal exudate cells from serotype I FIPV infected cats in the present study, further supporting its non-essential role in FIPV infection of monocytes and macrophages.

There is evidence that serotype I strains of FIPV may use DC-SIGN to bind and infect cultured CRFK cells [46]. Van Hamme et al. [45] also found that blocking of DC-SIGN inhibited binding of serotype I FIPV to feline monocyte cultures by one-third and infection by 80%. However, DC-SIGN mRNA was significantly under-expressed in the peritoneal exudate cells from cats with FIP, making it an unlikely receptor for in vivo infection. A related lectin, L-SIGN (CD209L), has been shown to be the receptor for the closely related human coronavirus 229E [47]. L-SIGN is expressed at high levels by hepatic cells [48] and is a high affinity receptor for hepatitis C virus [48]. An mRNA for L-SIGN was not detected in the present study. Likewise, the mRNA for ACE2 was also not detected, while the mRNA for dipeptidyl peptidase 4 was upregulated, but below the level of the study cutoff of plus or minus Log_2_FC. The down-regulation of DC-SIGN mRNA was unexpected, as it functions as an attachment receptor for enveloped viruses such as hepatitis C virus [48] by a process known as cis-infection. Trans-infection of dendritic cells by cytomegalovirus, whereby virus infection goes from one cell type to another, has also been associated with DC-SIGN [49]. HIV-1 also uses DC-SIGN and L-SIGN expressed by antigen presenting cells for its delivery to target cells such as CD4^+^ lymphocytes by trans-infection [50]. DC-SIGN may also be important in the capture and internalization of these pathogens for processing and antigen presentation and not merely replication [51].

Is there possible significance for the unexpected and highly significant down-regulation of DC-SIGN expression by peritoneal cells from FIPV infected cats? A common feature of the pathogens mentioned above is that they all can cause chronic infections that last a lifetime. This persistence has been linked to how DC- and L-SIGN and pathogen interact. Hepatis C virus can misuse DC- and L-SIGN by certain mechanisms to escape lysosomal degradation [52]. This “misuse” of DC-SIGN is thought to circumvent antigen processing or alter TLR-mediated signaling and skew immune responses towards Th2 rather than Th1 [53]. A Th2 type immune response to infection with *S. mansoni* is associated with its persistence and soluble carbohydrate egg antigens can cause a switch towards a Th2-cell-mediated immune response [54]. Mycobacteria may also target DC-SIGN to suppress DC function and modulate immune responses [55]. Therefore, the paradoxical down-regulation of DC-SIGN in FIPV infected macrophages may be one explanation for FIPV persistence in those cats that fail to contain the virus within the first few days following infection [1].

### Expression of Fc receptor genes

Fc receptors are found on several cell types, including B cells, follicular dendritic cells, tissue macrophages, NK cells and polymorphonuclear leukocytes. They are generally considered to be low-affinity receptors for Ig in immune complexes. Fc receptors have been shown to be important regulators of immune responses [56]. Although there are several Fc receptors, FcγRIIIA (CD16a) was the only Fc receptor gene that was both detected and over-expressed to a noticeable level in peritoneal exudate cells from cats with FIP. The likely sources of FcγRIIIA mRNA were NK cells, monocyte/macrophages and neutrophils. FcγRIIIA is the receptor on NK cells responsible for antibody dependent cellular cytotoxicity (ADCC). An important, and yet to be confirmed role for FcγRIIIA, is as a means for macrophage infection by FIPV. There is evidence that FIPV antibody binding is involved in the internalization of FIPV by macrophages [6] and that this process involves an Fc-type receptor [7] as shown for flaviviruses [57].

### Differential expression of mRNAs involved in Th1 and Th2 immunity

The prevailing belief is that cellular immunity is key for FIPV immunity [28, 58] and that antibodies are disease promoting [8, 28]. It is also widely believed that innate host defenses are crucial in the initial control of an infection but that they are insufficient for ultimate pathogen clearance [59]. It follows that survival of pathogens in macrophages is due to inhibition of adaptive (presumably cellular) immunity [60]. However, evidence that cellular immunity is crucial for preventing FIP is sparse and largely indirect. PBMC or whole blood cultures from FIPV immune cats proliferate in response to live virus exposure in vitro [57] and produce IFNγ in response to T cell epitopes of the viral N protein [61]. Delayed-type hypersensitivity responses can be elicited in the palpebral mucosa [58] or skin [62] with FIPV antigen in immune cats. The occurrence of FIP is also much higher in FECV enzootic cats naturally infected with feline leukemia virus, a known T cell immunosuppressive retrovirus [63]. The most convincing evidence for the role of cellular immunity comes from mouse hepatitis virus (MHV) infection. MHV, like FECV/FIPV, is a coronavirus that exists in enteric (wildtype) and systemic (mutated by laboratory passage) biotypes [64]. Infection of IFNγ deficient mice with systemic MHV caused a disease virtually identical to FIP [65]. It is also noteworthy that enteric MHV infection of B cell deficient mice causes a more prolonged intestinal infection, while the same virus in T cell deficient mice causes an FIP-like disease like what occurs when systemic MHV is inoculated into IFNγ deficient mice [66]. This suggests that FECV infection is controlled more by B-cell immunity, while FIPV infection is controlled by T-cell immunity.

An imbalance in T cell versus B cell immune responses by macrophages during the earliest stages of infection has been evoked as one reason for the inability of certain cats to resist FIP. One study demonstrated that the ratio of peripheral blood surface immunoglobulin positive cells (sIg^+^) to CD21+ cells was higher in cats with FIP than in naive cats, and that cells strongly expressing mRNA of the plasma cell master gene encoding B lymphocyte-induced maturation protein 1 (Blimp-1 or PRDM1), IL6, CD40 (TNFSF5) and (BAFF/TNFSF13B) were present in the abdominal effusions [67]. The mRNAs for all four of these genes were also significantly over-expressed by peritoneal exudate cells from cats with FIP in the present study.

It was hoped that differential expression levels of genes involved in Th1 and Th2 pathways would help demonstrate an imbalance towards Th2 immunity. On gross analysis, Th1 pathway genes appeared to have a higher level of over-expression than those in the Th2 pathway. However, viruses, bacteria and TLR ligands interact with antigen presenting cells and enter the Th1 differentiation pathway through DLL and it is noteworthy that DLL1 was significantly down-regulated as was the receptor NOTCH3. Evidence that favored Th2 pathway activation was not apparent from analysis of KEGG selected genes. Deficient Th2 associated polarization became less obvious when IL6, CD40, BAFF and Blimp-1 (PRDM1) genes were added to the list of KEGG Th2 differentiation genes. The mRNAs for all four of these genes were expressed at very high levels in peritoneal exudate cells from cats with FIP, confirming earlier studies of Takano et al. [68]. IL6 type cytokines are involved in inflammation and B cell maturation and inhibit IFNγ responses. CD40L expressed by T cells can provide signals to B cells that induce proliferation, immunoglobulin class switching, antibody secretion and prevention of apoptosis during B cell differentiation [67]. BAFF is expressed in B cell lineage cells and plays an important role in the proliferation and differentiation of B cells [69]. Blimp-1 (PDMR1) controls many functions of plasma cells, including migration and adhesion of plasmablasts, silencing B-cell specific gene expression associated with antigen presentation and class-switch recombination, and activating genes involved in antibody secretion [70].

In conclusion, the RNA-seq data obtained in this study has added to our knowledge of the flamboyant cytokine responses by peritoneal exudate cells from cats with experimentally induced FIP. In addition, it is hoped that the findings reported in this study, as well as additional data provided (NCBI database PRJNA342639), will prove useful to others in identifying specific areas for future studies on this important infectious disease of felids.
